# Time‐course of effects of external beam radiation on [^18^F]FDG uptake in healthy tissue and bone marrow

**DOI:** 10.1120/jacmp.v9i3.2747

**Published:** 2008-06-23

**Authors:** Adam L Kesner, Victoria K Lau, Michael Speiser, Wei‐Ann Hsueh, Nzhde Agazaryan, John J DeMarco, Johannes Czernin, Daniel HS Silverman

**Affiliations:** ^1^ Department of Molecular and Medical Pharmacology David Geffen School of Medicine University of California Los Angeles USA; ^2^ Department of Radiation Oncology David Geffen School of Medicine University of California Los Angeles USA

**Keywords:** Radiation therapy, treatment monitoring, small animal PET, FDG

## Abstract

The utility of PET for monitoring responses to radiation therapy have been complicated by metabolically active processes in surrounding normal tissues. We examined the time‐course of [^18^F]FDG uptake in normal tissues using small animal‐dedicated PET during the 2 month period following external beam radiation. Four mice received 12 Gy of external beam radiation, in a single fraction to the left half of the body. Small animal [^18^F]FDG‐PET scans were acquired for each mouse at 0 (pre‐radiation), 1, 2, 3, 4, 5, 8, 12, 19, 24, and 38 days following irradiation. [^18^F]FDG activity in various tissues was compared between irradiated and non‐irradiated body halves before, and at each time point after irradiation. Radiation had a significant impact on [^18^F]FDG uptake in previously healthy tissues, and time‐course of effects differed in different types of tissues. For example, liver tissue demonstrated increased uptake, particularly over days 3–12, with the mean left to right uptake ratio increasing 52% over mean baseline values (p<0.0001). In contrast, femoral bone marrow uptake demonstrated decreased uptake, particularly over days 2–8, with the mean left to right uptake ratio decreasing 26% below mean baseline values (p=0.0005). Significant effects were also seen in lung and brain tissue. Radiation had diverse effects on [^18^F]FDG uptake in previously healthy tissues. These kinds of data may help lay groundwork for a systematically acquired database of the time‐course of effects of radiation on healthy tissues, useful for animal models of cancer therapy imminently, as well as interspecies extrapolations pertinent to clinical application eventually.

PACs Number: 87.50.‐a

## I. INTRODUCTION

Positron‐emission tomography (PET) is an expanding, non‐invasive imaging technique frequently used for evaluating oncologic disease.^(1)^ It complements more conventional radiologic imaging techniques (i.e., CT and MRI), by looking at the functional or metabolic properties of suspected or confirmed tumor sites. More recently, evidence has also shown that fusion imaging with PET/CT significantly improves staging accuracy when compared to PET or CT alone.^2^, ^3^ Of the various radiotracers used for clinical indications, ^18^F‐fluorodeoxyglucose ([^18^F]FDG) is the most widely employed. [^18^F]FDG uptake, often quantified as a standardized uptake value (SUV), has been shown to be elevated in many types of cancers relative to normal tissues.^(4)^

In a recent review of the literature, Juweid et al. summarized how monitoring cancer treatment with PET contributed to tailoring an appropriate therapy regimen.^(1)^ In many studies, early metabolic changes measurable by [${}^{18}F$]FDG‐PET were highly predictive of clinical responses observed weeks to months later. Such findings have been reported for a variety of cancers, including lymphoma, as well as breast, esophageal, gastric, colorectal, head and neck, and non‐small‐cell lung cancers.^5^, ^16^ Early declines in [${}^{18}F$]FDG uptake generally correlate with longer progression‐free and overall survival. The available data suggest that [${}^{18}F$]FDG may be utilized for predicting treatment responses as early as one to three weeks after the first cycle of chemotherapy in a variety of cancer types.^8^, ^9^, ^15^, ^16^ This can prevent the exposure of patients to prolonged, ineffective treatments with undesirable side effects.

Metabolic activity in tumors also often decreases after successful radiation therapy.^17^, ^18^ However, the ability of [${}^{18}F$]FDG‐PET in monitoring the effects of radiation treatment has not been firmly established. This is in part due to the problem that, although [${}^{18}F$]FDG is an effective tumor‐localizing tracer, it is not tumor‐specific: benign processes (e.g., surrounding inflammatory changes, bone marrow suppression and hyperplasia) associated with irradiation can also alter [${}^{18}F$]FDG uptake levels. Hautzel et al. provided preliminary evidence of radiation‐related inflammatory changes contributing to initial enhancement of [${}^{18}F$]FDG uptake by assessing the metabolism of cervical lymph node metastases in a cancer patient during radiotherapy.^(17)^ They reported that low‐dose irradiation enhanced tumor glucose uptake, while higher doses were associated with subsequent metabolic decline. More recently, Metser et al., in a systematic review of PET/CT studies performed on oncologic patients during a 6‐month period, discovered benign non‐physiological uptake of [${}^{18}F$]FDG in more than 25% of the studies. In half of these, [${}^{18}F$]FDG uptake was comparable to that of malignant sites, and most of the benign lesions were inflammatory in nature.^(19)^

Differentiation of inflammatory processes from residual or recurrent disease is complicated, leading to imaging pitfalls such as false‐positive readings and consequently, administration of unnecessary therapy. Data from several recent studies suggest that PET can remain relatively nonspecific for up to 6 months following radiation therapy, due to inflammatory changes which may occur in the first few months after treatment.^(20)^

In a field where treatment regimens often have success rates falling below fifty percent, improved methods for accurate, early prediction of treatment failure would be of substantial clinical value. The purpose of this study was to longitudinally characterize and quantify the time‐course of [${}^{18}F$]FDG uptake in a variety of healthy tissues, occurring subsequent to irradiation, under experimentally controlled conditions, through the use of non‐invasive imaging with small animal‐dedicated PET.

## II. MATERIALS AND METHODS

### A. Irradiation

All animal studies were performed under a protocol approved by the Chancellor's Animal Research Committee of UCLA. Four male mice (strain C57BL/6) underwent microPET/CT imaging. PET images were acquired on a microPET Focus 220 (Siemens Medical Solutions, Malvern, PA) and CT images were acquired on a MicroCAT II (Imtek Inc., Knoxville, TN). Small animal PET and CT scans were acquired one hour after intravenous administration of 7.5 MBq (0.2 mCi) [${}^{18}F$]FDG on days 0 (pre‐radiation), 1, 2, 3, 4, 5, 8, 12, 19, 24, and 38. Each mouse was irradiated with 12 Gy of external beam radiation (max. dose), in a single fraction to the left half of the body.

**Figure 1:**
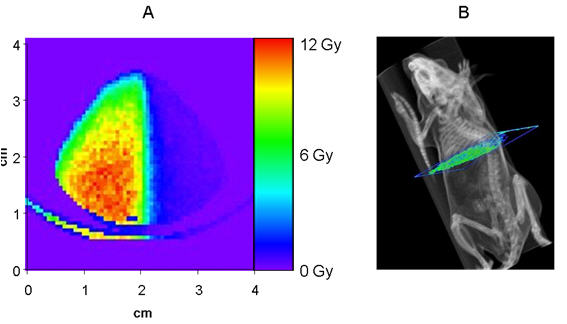
(A) Monte‐carlo estimate of dose distribution for a mouse receiving radiation to the left half of the body from a 6 mV linear accelerator. The distribution represents an axial slice of the mouse, just inferior to the lungs. (B) Illustration portraying the location of the dose calculation shown in figure.

Since the mice used in this experiment were small (approximately 2 cm in width across the thorax), great care was taken to deliver a dose distribution to provide a sharp dose falloff from the left side of the mouse to the right. A dedicated 6MV Novalis radiosurgery LINAC (BrainLAB, Gmbh, Germany) was used to deliver a posterior/anterior beam with a half‐beam block. Additionally, a lead jig was created and placed directly above the mouse to further reduce the beam's penumbra and subsequent dose received by the right half of the body. Film dosimetry of the resulting field and the treatment planning system's calculations were used to assess the dose falloff and determine required monitor units for a maximum point dose of 12 Gy. Additionally, a Monte Carlo simulation, using a model of the Novalis LINAC and a micro CT of one of the mice, was used to assess and quantify the resulting relative dose distribution in the irradiated mice. Resultant dosimetry from the Monte Carlo simulation is depicted in Fig. 1, for an axial slice of a mouse CT scan. Metabolic activity, assessed with [${}^{18}F$]FDG small animal PET, in various tissues (i.e., lungs, femoral bone marrow, brain, and liver), was compared with the irradiated left and non‐irradiated right body halves before, and at each time point after, external beam radiation.

## B. PET Acquisition

**Figure 2:**
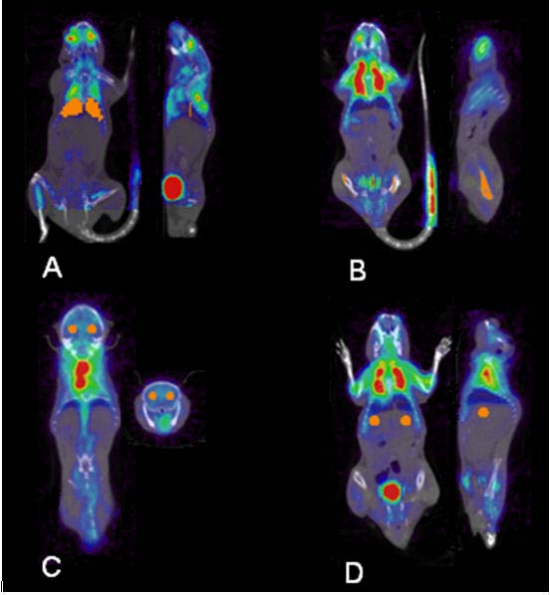
Display of hand‐drawn ROIs (displayed in orange) for lungs (A), femur (B), brain (C), and liver (D). For each area assessed, ROI's were drawn using the Amide software package, and the uptake in the irradiated left tissue was compared with uptake in non‐irradiated contralateral tissue.

In this study, a total of 44 small animal PET and CT scans were acquired from four different mice. MicroPET/CT images were reconstructed using a filtered back projection algorithm (ramp filter, voxel size $0.04\times 0.04\times 0.0796\;{\text{cm}}^{3})$, and the biodistribution of [${}^{18}F$] FDG was assessed in regions of interest (ROIs) with use of the Amide software package (freeware available at http://amide.sourceforge.net). Uptake in irradiated tissue was compared with uptake in non‐irradiated tissues. ROIs were obtained for left and right portions of each tissue assessed: lungs, femur, brain, and liver (Fig. 2). Ratios of left to right uptake in ROIs were calculated for each mouse, for all trial days within the two‐month study period, by a single rater, to eliminate inter‐observer variability.

### C. Statistical Analysis

**Figure 3:**
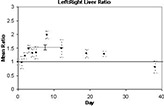
Time‐course of mean [${}^{18}F$]FDG uptake ratio in liver. Each data point represents the mean left:right ratio of uptake values calculated for four mice. The ± standard error is indicated with dashed bars. A thick grey line corresponds to the mean for the range of dates indicated in Table 1 (days 3–12 for liver).

**Figure 4:**
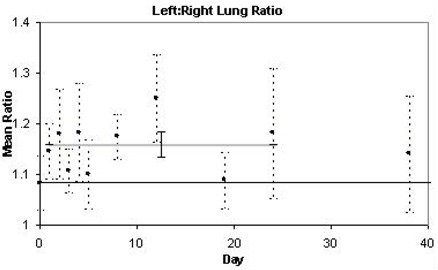
Time‐course of mean [${}^{18}F$]FDG uptake ratio in lungs. Each data point represents the mean left:right ratio of uptake values calculated for four mice. The ± standard error is indicated with dashed bars. A thick grey line corresponds to the mean for the range of dates indicated in Table 1 (days 1–24 for lungs).

**Figure 5:**
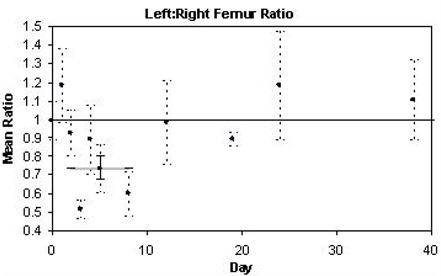
Time‐course of mean [${}^{18}F$]FDG uptake ratio in femur. Each data point represents the mean left:right ratio of uptake values calculated for four mice. The ± standard error is indicated with dashed bars. A thick grey line corresponds to the mean for the range of dates indicated in Table 1 (days 2–8 for femur).

**Figure 6:**
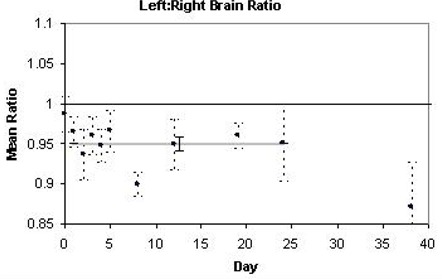
Time‐course of mean [${}^{18}F$]FDG uptake ratio in brain. Each data point represents the mean left:right ratio of uptake values calculated for four mice. The ± standard error is indicated with dashed bars. A thick grey line corresponds to the mean for the range of dates indicated in Table 1 (days 1–24 for brain).

Time activity curves were examined for four organs, using the 11 scans acquired for each animal. Time windows used for statistical analysis were chosen by qualitatively selecting periods where a relatively consistent separation in the left‐to‐right ratios, relative to baseline data, were apparent on visual interpretation of time‐course data (as reflected in Figs. 3–6 and in the fifth column of Table 1).

**Table 1:** Summary of mean [${}^{18}F$]FDG uptake ratios observed in four different types of tissue. Irradiation had varying effects on [${}^{18}F$]FDG uptake in previously healthy tissues.

*Tissue*	*Direction of peak change*	*Time to peak change*	*Magnitude of peak change*	*Period of most apparent effect of irradiation*	*Average magnitude of change during noted period*	*p‐value (two‐tailed)*
Liver	↑	8 days	100%	Days 3–12	52%	<0.0001
Lungs	↑	12 days	15%	Days 1–24	7%	0.0127
Femur	↓	8 days	40%	Days 2–8	26%	0.0005
Brain	↓	8 days	10%	Days 1–24	5%	<0.0001

Relative uptake values in the analyzed time windows, reported as left to right uptake ratios for each area evaluated, were statistically assessed for significance by use of two‐tailed Student's *t*‐tests. Response patterns of [${}^{18}F$]FDG uptake in the liver, lungs, bone marrow of the femur, and brain were assessed. At baseline, no significant differences in uptake were found between left and right‐sided tissues prior to irradiation (left:right ratios were 1.00±0.10, 1.08±0.05, 1.00±0.10, and 0.99±0.02, mean ± SE for liver, lungs, bone marrow, and brain, respectively). Significance of changes in left to right ratio from 1 was assessed for times subsequent to administration of 12 Gy external beam radiation.

## III. RESULTS

Observed as early as the first day, irradiation had a significant impact on [${}^{18}F$]FDG uptake in previously healthy tissues (Table 1). The time‐course of these effects differed dramatically, depending on the type of tissue examined (Figs. 3–6), with the percentage differences of left to right ratios relative to baseline increasing or decreasing from 5% to over 50%.

### A. Liver

Irradiation of the left liver resulted in higher [${}^{18}F$]FDG uptake than in the non‐irradiated right side. This effect peaked on day 8, when the left to right ratio was 100% greater than at baseline (p<0.0001), and was most apparent on days 3–12, over which time the left to right ratio averaged 52% higher than at baseline (p<0.0001). Fig. 3 illustrates the time‐course of these effects, with each data point representing the mean [${}^{18}F$]FDG uptake in four mice on each scan day.

### B. Lungs

Irradiation also resulted in higher [${}^{18}F$]FDG uptake in the irradiated left lung compared to the non‐irradiated right lung. This rise in the mean left to right uptake ratio was observed as early as day 1, and peaked on day 12, before returning to baseline levels. Fig. 4 illustrates the time‐course of these effects within a 2 month period. The change in mean left to right uptake ratio post‐irradiation was found to be statistically significant, resulting in a rise of 16% relative to baseline, averaged over days 1–24 (p<0.0001). It is noteworthy that at baseline, the lungs demonstrated slightly higher uptake in the left lung relative to the right, most likely due to cardiac spillover. Thus, each mouse was also statistically analyzed after being normalized to its own baseline, and results remained significant, resulting in an increase in ratio of 7% (p=0.01).

### C. Femur

Irradiation decreased the mean left to right uptake ratio in the femur, which was most prominent on trial days 2–8. The most significant decrease was observed on day 8, when uptake was 40% below baseline values (p<0.05). Fig. 5 illustrates the time‐course of these effects over a 2 month period. Over days 2–8, the left to right ratio averaged 26% lower than at baseline (p=0.0005).

### D. Brain

As observed in the femur, irradiation decreased the mean left to right uptake ratio in the brain, which was observed on all trial days post‐irradiation, again most significant on day 8. Fig. 6 illustrates the change in [${}^{18}F$]FDG uptake in the irradiated left brain compared to that of the non‐irradiated right brain, resulting in a 5% decrease relative to baseline, averaged over post‐radiation days 1–24 (p<0.0001).

## IV. DISCUSSION

In the present study, we systematically documented the direction, magnitude, and time‐course of radiation‐induced changes occurring in a variety of tissue types. While the irradiated liver and lungs demonstrated increases in [${}^{18}F$]FDG uptake in the days following irradiation, irradiated femoral bone marrow and brain demonstrated decreases in [${}^{18}F$]FDG uptake during that period. Effects ranged from 5% to over 50% changes in uptake relative to the pre‐irradiated baseline, and each tissue type exhibited a distinct time‐course of uptake over a two month trial period.

In the femur and brain, we observed decreases in the irradiated/non‐irradiated tissue uptake ratios following radiation. The declining uptake in the femur is understandable in the context of previously documented responses ^21^, ^23^ that bone marrow is highly sensitive to radiation, and decreased [${}^{18}F$]FDG uptake may be a result of functional suppression following radiation. In the brain, decreased [${}^{18}F$]FDG uptake is also not surprising, given that the immune system has less access to brain tissue than to the lung and liver and other tissues, due to the blood‐brain barrier,^24^, ^26^ coupled with a normally high rate of glucose metabolism which occurs in the brain at baseline,^27^, ^28^ and which can be disrupted by the synaptic dysfunction occurring subsequent to irradiation.

In the lungs and the liver we observed an increase in the irradiated/non‐irradiated uptake ratios following radiation. This increase most likely results from an inflammatory response in these tissues.^29^, ^30^ Specifically, early inflammation in the lung may stem from the immediate expression of the pro‐inflammatory cytokines TNF‐alpha, IL‐1 alpha, and IL‐6 in the bronchiolar epithelium in the first hours after lung irradiation.^(31)^ In the liver, the high levels of inflammation may result from high levels of oxidative stress, as reflected in some studies by elevated levels of peroxidative damage, DNA fragmentation, LDH activity, and nitric oxide levels.^(32)^

We have characterized the time‐course of effects of radiation in various healthy tissues from which cancer may arise. Although [${}^{18}F$]FDG‐PET is commonly employed for monitoring responses to chemotherapy,^8^, ^9^, ^15^, ^16^ it has been less utilized in monitoring effects following irradiation. While the exact mechanisms and extent of metabolic responses in healthy tissues have not yet been well defined, interpretation of [${}^{18}F$]FDG uptake can be substantially complicated by radiation‐induced inflammation and other effects occurring in surrounding tissues. As discussed by Engenhart et al., it is often difficult to distinguish the difference in [${}^{18}F$]FDG uptake before and after irradiation, as it does not reliably differentiate among proliferation, repair, inflammation, and residual viable tumor cells in patients with inoperable recurrent rectal carcinoma.^(33)^ Data established in the present study may be placed in the context of other published studies that have investigated irradiation effects. Ohtsuka et al. investigated non‐small‐cell lung cancer after neoadjuvant chemoradiotherapy, and found positive [${}^{18}F$]FDG uptake in PET scans despite absence of tumor cells found pathologically.^(34)^ Such false positives are thought to be due to either inflammatory lesions with invasion of macrophages and lymphocytes resulting in increased uptake of [${}^{18}F$]FDG,^35^, ^37^ or metaplastic and proliferative epithelial elements caused by chemoradiotherapy leading to [${}^{18}F$]FDG accumulation.^(38)^ Similarly, in our study, [${}^{18}F$]FDG PET demonstrated increased metabolic activity in the liver and lungs. However, not all research has found significance in the post irradiation changes in PET in the organs we looked at. Castellucci et al. investigated the rate of postactinic inflammatory alterations leading to potential false‐positive PET images in lymphoma patients with the hope of determining an optimal time window between radiation therapy and [${}^{18}F$]FDG‐PET. They found that the incidence of inflammation shortly after radiation therapy was not as prevalent as they had expected, and they were unable to establish a strong link to the elapsed time since the end of radiation therapy treatment.^(39)^ More research is clearly needed in this area.

In summary, results from our present study indicated effects of tissue irradiation ranging from 5% to over 50% changes in uptake relative to the pre‐irradiated baseline, with different tissue types exhibiting distinct time‐courses of uptake over a two‐month observation period. Limitations to our study include the difficulty of administering a uniform radiation dose across mice, as dose depends on size, shape, and composition of the irradiated subject as well as technical parameters of the linear accelerator. To account for this, we classified dose distribution by using a Monte Carlo simulation, which utilizes a computer model to make iterative predictions about how the radiation was able to be delivered, especially for the left to right comparison. We used mice of the same body weight and age for our study, to obtain as homogenous an effect of irradiation as possible.

It is also important to recognize that different doses and forms of irradiation may yield different time‐courses of post‐radiation effects. What our results may provide is initial insight into the relative magnitudes of biological effects following irradiation. These preliminary findings of the diverse effects of irradiation in healthy tissues could be useful for animal models of cancer therapy (e.g., xenograft models) and provide a point of reference for further studies aimed at trying to delineate and quantify uptake in tumors and their associated tumor to background ratios. Actual rates of metabolism will also need to be established in humans, as it is common for physiological and pathological processes to be accelerated in mice relative to normal reactions in people.^(40)^ Translating these processes to the clinic can potentially aid in the differentiation of inflammatory processes from that of residual or recurrent disease. In PET, lesion characterization is often heavily dependant on lesion background uptake ratio. Recent literature ^18^, ^41^, ^42^ has suggested that a 20% change in this ratio is clinically significant. However, lesion detection can depend on differences ranging within a few percent. Thus the extent to which radiation impacts this ratio can have direct implications on clinical diagnosis. Both PET and radiation are largely utilized clinically, and further study may expand the role of PET for radiation treatment monitoring, as it is currently starting to be explored in the clinic.^43^, ^45^ Examining other radiotracers with this experimental design is also of interest, as different radiotracers may behave differently, during radiotherapy.^27^, ^46^

## V. CONCLUSIONS

Different tissues have different metabolic profiles with respect to the direction, magnitude, and time‐course of changes occurring after irradiation. We saw increased FDG uptake following radiation in the lungs and liver, while we noticed the opposite effect in the brain and femur. Time courses and rates of reactions varied among these tissues, likely reflecting the variety of biological processes encountered when combining radiation treatment with FDG PET imaging. Data from studies such as this one may help in designing animal models of monitoring tumor responses to irradiation imminently, as well as, ultimately, in translating the findings to optimizing clinical therapeutic monitoring.
